# A Digital Human for Delivering a Remote Loneliness and Stress Intervention to At-Risk Younger and Older Adults During the COVID-19 Pandemic: Randomized Pilot Trial

**DOI:** 10.2196/31586

**Published:** 2021-11-08

**Authors:** Kate Loveys, Mark Sagar, Isabella Pickering, Elizabeth Broadbent

**Affiliations:** 1 Department of Psychological Medicine The University of Auckland Auckland New Zealand; 2 Auckland Bioengineering Institute The University of Auckland Auckland New Zealand; 3 Soul Machines Ltd Auckland New Zealand

**Keywords:** COVID-19, loneliness, stress, well-being, eHealth, digital human, conversational agent, older adults, chronic illness

## Abstract

**Background:**

Loneliness is a growing public health issue that has been exacerbated in vulnerable groups during the COVID-19 pandemic. Computer agents are capable of delivering psychological therapies through the internet; however, there is limited research on their acceptability to date.

**Objective:**

The objectives of this study were to evaluate (1) the feasibility and acceptability of a remote loneliness and stress intervention with digital human delivery to at-risk adults and (2) the feasibility of the study methods in preparation for a randomized controlled trial.

**Methods:**

A parallel randomized pilot trial with a mixed design was conducted. Participants were adults aged 18 to 69 years with an underlying medical condition or aged 70 years or older with a Mini-Mental State Examination score of >24 (ie, at greater risk of developing severe COVID-19). Participants took part from their place of residence (independent living retirement village, 20; community dwelling, 7; nursing home, 3). Participants were randomly allocated to the intervention or waitlist control group that received the intervention 1 week later. The intervention involved completing cognitive behavioral and positive psychology exercises with a digital human facilitator on a website for at least 15 minutes per day over 1 week. The exercises targeted loneliness, stress, and psychological well-being. Feasibility was evaluated using dropout rates and behavioral observation data. Acceptability was evaluated from behavioral engagement data, the Friendship Questionnaire (adapted), self-report items, and qualitative questions. Psychological measures were administered to evaluate the feasibility of the trial methods and included the UCLA Loneliness Scale, the 4-item Perceived Stress Scale, a 1-item COVID-19 distress measure, the Flourishing Scale, and the Scale of Positive and Negative Experiences.

**Results:**

The study recruited 30 participants (15 per group). Participants were 22 older adults and 8 younger adults with a health condition. Six participants dropped out of the study. Thus, the data of 24 participants were analyzed (intervention group, 12; waitlist group, 12). The digital human intervention and trial methods were generally found to be feasible and acceptable in younger and older adults living independently, based on intervention completion, and behavioral, qualitative, and some self-report data. The intervention and trial methods were less feasible to nursing home residents who required caregiver assistance. Acceptability could be improved with additional content, tailoring to the population, and changes to the digital human’s design.

**Conclusions:**

Digital humans are a promising and novel technological solution for providing at-risk adults with access to remote psychological support during the COVID-19 pandemic. Research should further examine design techniques to improve their acceptability in this application and investigate intervention effectiveness in a randomized controlled trial.

**Trial Registration:**

Australia New Zealand Clinical Trials Registry ACTRN12620000786998; https://www.anzctr.org.au/Trial/Registration/TrialReview.aspx?id=380113

## Introduction

This study investigated the feasibility and acceptability of a digital human (DH) that delivered a psychological intervention to mitigate the effects of social restrictions on loneliness, stress, and well-being in vulnerable populations during the COVID-19 pandemic. The results will inform the design of a randomized controlled trial (RCT) to evaluate intervention effectiveness. To provide a rationale and context for the study, the introduction describes the effects of the COVID-19 pandemic on loneliness, the importance of treating loneliness, and previous work on robot and conversational agent (CA) interventions for loneliness.

Many countries have adopted socially restrictive public health measures over recent months to slow the spread of the COVID-19 pandemic, including the United Kingdom, Canada, the European Union, Japan, and Australia [1]. Precautions have included bans on mass gatherings, closure of schools and businesses, mandatory work from home conditions, and limits on social activities [2]. New Zealand adopted some of the strictest lockdown rules globally, which included 2-m physical distancing between people and staying at home, except for essential trips to a supermarket or pharmacy, or to seek medical care, with restrictions gradually eased as appropriate [3]. Individuals who were at risk of developing a severe illness should they contract COVID-19 were advised to take additional precautions to social distance and isolate [4]. This included older adults over the age of 70 years (who are at greater risk of dying from COVID-19) [5] and younger adults with an underlying medical condition who may be immunocompromised [6].

While these precautions can help protect vulnerable populations, there are mental health implications of strict social distancing, including increased loneliness [7]. Older adults and adults with underlying health conditions were already at greater risk of loneliness prepandemic [8,9], and these restrictions have exacerbated this risk. Interventions to reduce loneliness are especially important for this group given the long-term implications for health as described below.

Loneliness is a subjective psychological state in which a person perceives a mismatch between their actual and desired social relations [10]. While brief feelings of loneliness can serve as an adaptive motivator to seek social interaction, chronic loneliness has negative effects on physical and mental health outcomes [11,12]. Loneliness is associated with feelings of stress [13], which activate the body’s “fight or flight” response. The sympathetic nervous system becomes activated, and over a prolonged period, it creates negative downstream effects on the cardiovascular, neuroendocrine, and immune systems [14]. As a result, chronic loneliness has been associated with increased risks of morbidity (eg, coronary heart disease, high blood pressure, and stroke) [11] and mortality [15]. Loneliness can be improved through psychological interventions that target the following 4 key areas: changing maladaptive social cognitions, increasing social support, increasing opportunities for social interaction, and improving social skills [16].

Loneliness interventions can be delivered in-person or remotely through technology, and both have been shown to be effective [16], including for older adults [17]. In-person loneliness interventions have included individual psychotherapy involving social cognitive training as part of cognitive behavioral therapy [18], mindfulness-based therapies [19], and social support groups [20]. However, remote interventions may be more suitable for at-risk individuals in isolation as a result of the pandemic. Remote therapies for loneliness have included internet-based cognitive behavioral therapy [21] and internet skills training to access online support [22]. However, research has shown that engagement with technology-based interventions is often lower outside of a clinical trial context [23,24].

Artificial agents may be a particularly engaging way to provide psychological support to people during a pandemic. People have been shown to feel a sense of social presence with artificial agents, which can improve technology engagement [25,26]. Social robots are artificial agents with embodiment in a physical hardware form that are capable of social interaction and are programmed to autonomously interact with their physical environment [27]. CAs are artificial agents that include a dialogue system, and may or may not include a digital embodiment or face [28]. Under the umbrella term of CAs fall chatbots, embodied CAs, voice assistants, and DHs, among others. CAs may be more feasible for providing remote support than robots because they are less expensive and more scalable [29], as they can be accessed through websites or software applications on devices that many patients already own (eg, smartphones and computers).

A recent scoping review on robot-facilitated loneliness interventions found evidence supporting their use with older adults [30]. For example, Paro, a companion robot in the form of a fluffy baby harp seal, alleviated feelings of loneliness in older adults in nursing homes by providing direct companionship in a manner akin to a pet [31]. Other robots include Giraff (a telepresence robot that connects users and their families over video call [32]), MARIO (which includes a My Memories function where users can show photographs to others as a conversation starter [33]), and SYMPARTNER (which reminds people of their upcoming social engagements [34]). Social robots may also improve loneliness in younger adults [35]. Robots have been shown to be effective at delivering other kinds of psychological interventions, such as positive psychology interventions for well-being [36].

Research looking at the clinical effectiveness of CAs in health care is relatively limited, and a more robust methodology is required [28]. However, a study found that daily conversations with an animal-like embodied CA over the course of a hospital stay significantly improved loneliness in older adults [37]. Another study found that daily interactions over 1 week with a human-like embodied CA that used a proactive communication strategy improved loneliness and happiness in older adults [38].

CAs also show promise for delivering psychological therapies to improve stress and well-being; outcomes that may be worsened by chronic loneliness. Vivibot, a Facebook messenger chatbot that delivered positive psychology exercises over 4 weeks, was found to be acceptable and effective for reducing anxiety in young adults with a chronic health condition [39]. Other research has found that a Facebook messenger chatbot that delivered cognitive behavioral therapy exercises, such as mindfulness and gratitude activities, improved stress and well-being [40].

DHs are a new type of CA that use artificial intelligence to build social and emotional engagement with users [41], which could help to reduce loneliness. DHs differ from other CAs in that they are modeled off real people using Hollywood light room technology and computer-generated imagery (CGI) animation techniques [42]. This provides DHs with a very life-like appearance. In addition, DHs include a complex cognitive architecture modeled off humans and involve a digital brain with virtual neurotransmitters to influence behavior [43]. For example, while in “high oxytocin mode,” DHs show attachment and separation distress toward users, which can help to build a bond [44]. DHs use live neural networks while interacting with people to classify their emotional state, and respond to people using a combination of speech, facial behaviors, and body gestures. DHs may be a particularly promising technology to deliver a remote loneliness intervention given their engaging social abilities and scalability; all that users require to access one is a computer and an internet connection. However, as DHs are a relatively new technology, it is unknown whether they are a feasible and acceptable way to deliver a remote loneliness intervention.

This study aimed to investigate whether a DH was a feasible and acceptable method of delivering a remote loneliness and stress intervention to high-risk adults during the COVID-19 pandemic. In addition, this study evaluated the feasibility of the study methods in advance of a future definitive RCT. It was hypothesized that a DH would be a feasible and acceptable method of intervention delivery, and that the study methods would be feasible. The results will inform the design of an RCT to investigate the effectiveness of the DH intervention.

## Methods

### Trial Registration

This trial was reported in keeping with the CONSORT (Consolidated Standards of Reporting Trials) 2010 statement extension for randomized pilot and feasibility trials [45]. Ethics approval was obtained from the University of Auckland Human Participants Ethics Committee on July 06, 2020 (approval number: 024752). The trial was prospectively registered with the Australia New Zealand Clinical Trials Registry on August 04, 2020 (registration number: ACTRN12620000786998).

### Trial Design

A randomized pilot trial was conducted involving a parallel mixed design with a waitlist control condition (1:1 allocation ratio). The primary outcomes were feasibility and acceptability, and the secondary outcomes were rapport with the DH, loneliness, stress, COVID-19 distress, positive and negative experiences, and psychological well-being. No major changes were made to the methods after commencing the trial.

### DH Intervention

**Figure 1:**
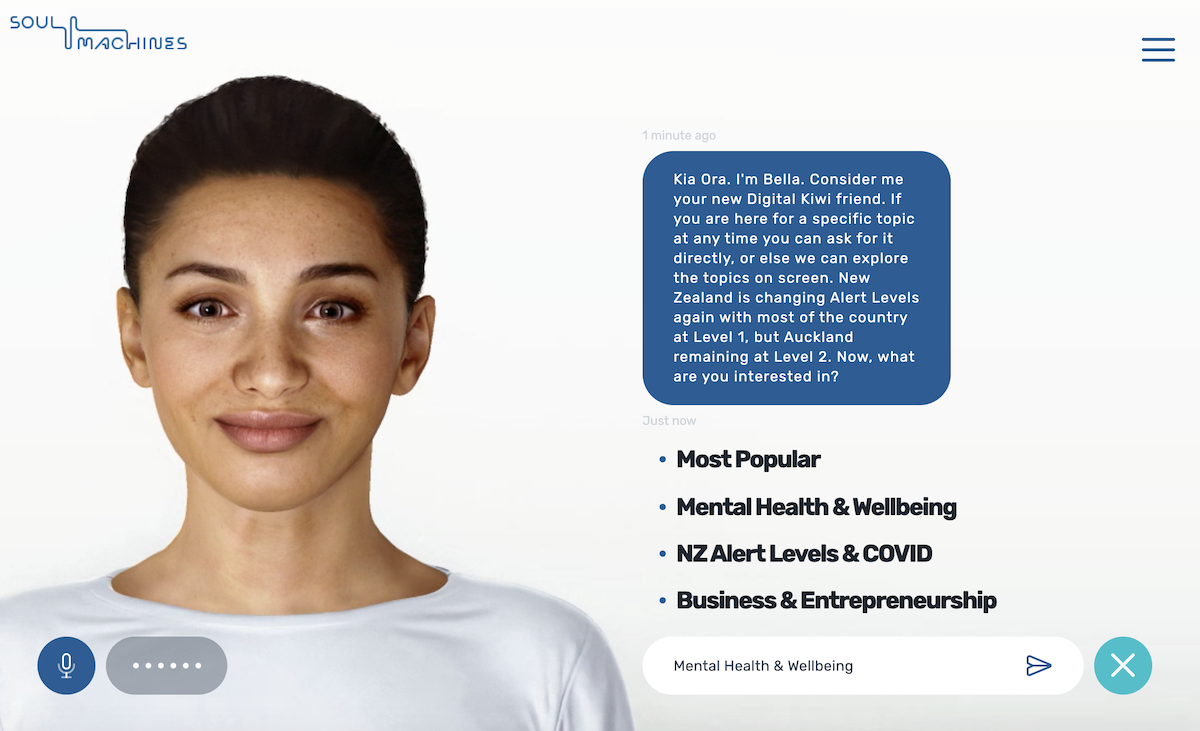
The digital human's user interface when using the messenger function.

The DH facilitator (“Bella”) was developed by Soul Machines Ltd (Auckland, New Zealand) (Figure 1). Bella was autonomously animated and presented on a website that participants accessed from their personal computer, tablet, or smartphone. Bella was modeled to be a young adult female of mixed race (Māori and New Zealand European). She was synthesized from the visual features of several human models (ie, not modeled off a singular person). Bella was presented in front of a white background in a portrait view of her head and shoulders. Her appearance, background, and proximity to the screen remained consistent throughout the study.

Bella autonomously responded to participants’ language using a finite state conversation engine with preprogrammed responses. Bella was programmed to have some autonomous variation in her language for phrases that would not affect her intervention delivery (eg, she varied her greetings each day). Bella spoke using a computer-generated female voice with an Australian accent (“Wavenet C – female” by Google). Participants could communicate with Bella in 1 of the following 3 ways: (1) speech, (2) typing, and (3) clicking on-screen buttons (where present). Bella always responded to participants in speech; however, if participants opened the messenger window to type, they could see a typed version of Bella’s speech as well (see Figure 1 for an example). If Bella did not understand a participant’s language, she would say, “I’m sorry, I didn’t understand. Could you please repeat or reword your statement?” or similar. If she did not understand after a couple of attempts, she would redirect the participant back to her main menu.

Bella engaged in human-like facial and body gestures as she spoke, including blinking, maintaining eye gaze, raising her eyebrows, and moving her head and shoulders. She showed emotional expressions on her face as she spoke to portray joy and concern, which were preprogrammed and triggered by phrases she spoke using text-to-speech emotional markup language. This involved a process of manually tagging language in her script to elicit particular facial emotions each time Bella spoke the phrase. Bella’s facial expressions were autonomously generated in real-time using visual computing and neurobehavioral modeling techniques (described in greater detail in previous reports [41-43,46]). Bella had a virtual nervous system that contained virtual neurotransmitters and live neural networks to process emotional data and inform her responses; however, these capabilities were not used in this study in order to maintain experimental control.

Bella was designed to deliver several relationship building strategies derived from psychology [47] and human-computer interaction research [48]. These included engaging in shared activities with the user, mutual self-disclosure, showing empathy, expressing the value of the friendship, and being nonjudgmental. These relationship building strategies were incorporated into Bella’s language at various points in the interaction.

Participants were informed that Bella continuously collected speech and video data in order to communicate (eg, to hear speech and to make eye contact). These data were not recorded, saved, or analyzed by the researchers. Bella’s data collection and use processes are in keeping with the European Union General Data Protection Regulation (GDPR) [49,50].

### DH Intervention Content

Participants were asked to prioritize visiting the mental health and well-being content that Bella offered as part of their daily website visit. This content included evidence-based exercises to improve loneliness, stress, and psychological well-being, as described below.

#### The Expressing Kindness Challenge

Three challenges were delivered over 3 days and included evidence-based strategies to improve loneliness and psychological well-being. The first 2 challenges were (1) to make contact with an old friend, relative, or someone the participant had not been in touch with for a while and (2) to contact someone to let them know something that the participant appreciated about them. These tasks aimed to increase opportunities for social interaction, strengthen social support, and improve social skills. The third challenge asked the participant to make a list of 3 things that they were grateful for, as a positive psychology exercise. Each of the challenges was accompanied by examples to help the participant generate ideas (eg, on day 2, Bella told participants something that she appreciated about them). At the end of the module, participants were reminded to continue practicing kindness toward others and themselves.

#### The Brain and Stress Module

**Figure 2:**
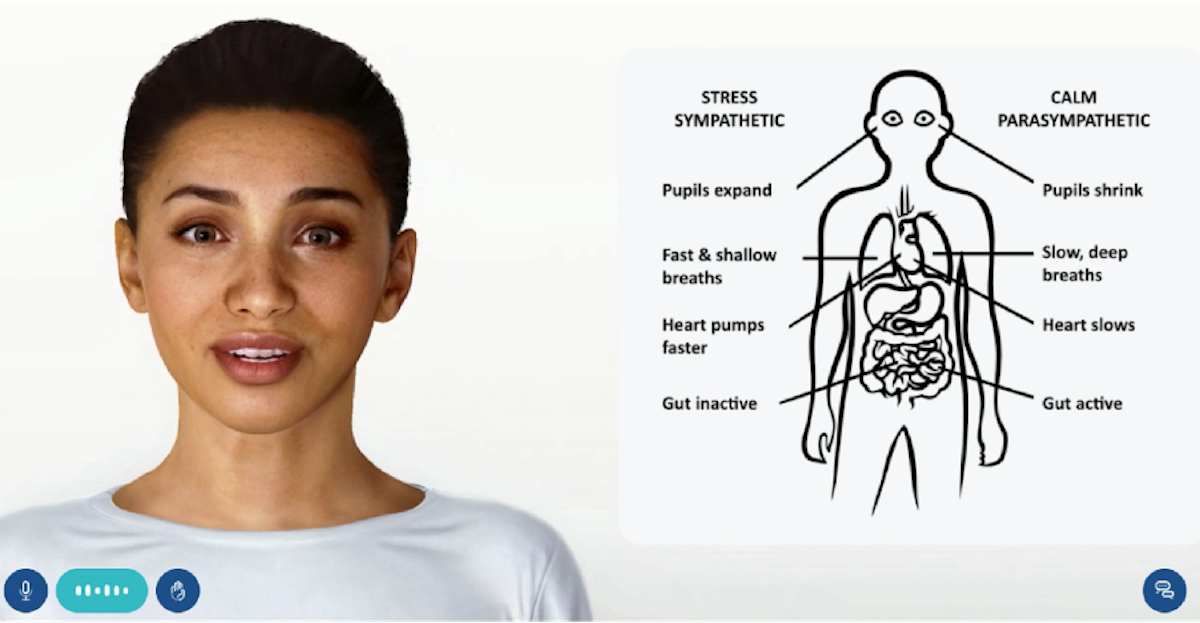
The digital human interface during psychoeducation as part of the brain and stress module.

This module provided psychoeducation about stress and stress awareness through verbal explanations and diagrams over 1 visit (Figure 2). It covered how stress affects the body and symptoms that are associated with the stress response. The module encouraged participants to reflect on the sources of stress in their lives, and informed participants of behavioral strategies for stress management. These included educating participants about a deep breathing exercise that they could practice, and linking participants to the Headspace website [51], where participants could access audio recordings of deep breathing and meditation exercises. At the end, participants were encouraged to visit the mental health tips, which are described in further detail below.

#### Mental Health Tips

Six modules each focused on a separate psychological well-being tip. The tips encouraged social connection, exercise, acknowledging feelings, being mindful of anxiety-provoking news media consumption, doing activities that elicit positive emotions, and trying out behaviors from a self-care guide.

#### Other Conversation Modules

Participants were able to talk with Bella about a range of other topics beyond mental health and well-being. This included information about the COVID-19 pandemic (eg, New Zealand’s alert levels, details about the virus, symptoms and prevention, and New Zealand’s Healthline and health support resources), and information regarding business and entrepreneurship (eg, remote work and business support organizations).

### Participants

Thirty participants were recruited. Participants were adults who were at greater risk of developing severe illness if they contracted COVID-19, and as a result, they were asked by the local New Zealand Government to self-isolate to a greater degree during the pandemic. They included (1) older adults aged 70 years or older and (2) adults aged 18 to 69 years who had at least one underlying medical condition that increased the risk of contracting severe COVID-19. The underlying medical condition could have included a serious respiratory disease (such as a chronic lung disease or moderate to severe asthma), a serious heart condition, an immunocompromised condition (such as cancer treatment, smoking-related illness, bone marrow or organ transplantation, hematologic neoplasms, immune deficiency, uncontrolled HIV or AIDS, and prolonged use of corticosteroids and/or other immune-weakening medications such as disease-modifying antirheumatic drugs), a BMI of 40 or higher, diabetes, chronic kidney disease, dialysis, liver disease, and/or pregnancy at the third trimester stage. Participants were required to have English fluency, and access to a computer and internet connection at home. Participants who were 70 years or older were required to achieve a score of 25 or higher on the Mini-Mental State Examination (MMSE). Potential participants were excluded if they received a score of 24 or lower on the MMSE, which would indicate cognitive decline to a moderate or greater degree. Participants aged 70 years or older were not excluded on the basis of whether or not they had an underlying health condition, as their age placed them at a higher risk of developing severe COVID-19.

Twenty-two older adult participants (aged 70 years or older) were recruited from 5 Summerset retirement village sites around the greater Auckland area. Recruitment methods involved presentations to residents about the research, email flyers, and caregiver word of mouth. Residents approached the research team if they were interested in participating. Eligibility screening involving the MMSE and an informed consent procedure (for those who were eligible) were conducted in-person at the retirement village with a member of the research team.

Eight younger adult participants (aged 18-69 years with an underlying medical condition) were recruited from a flyer posted to a staff email list at the University of Auckland, in addition to targeted Facebook advertising, word of mouth, and a Summerset retirement village presentation. Younger adults interested in taking part completed an eligibility screen and informed consent procedure online via a survey website (Qualtrics), except 1 participant who was recruited from a retirement village presentation. This participant completed an eligibility screen and informed consent procedure in-person.

A recruitment target of 30 participants was set, as a minimum of 12 participants per group is recommended for a feasibility study due to precision about the mean and variance [52], and to allow for 20% attrition. Recruitment took place between November 11, 2020, and March 04, 2021, with a 3-week break from late December. Recruitment stopped once the quota of 30 participants had been reached.

Data were collected from online questionnaires using Qualtrics, which participants completed from their place of residence. For older adult participants, this may have included completion from a Summerset retirement village independent living villa or apartment, or from the nursing home facility. For younger adult participants, participation took place online from their place of residence in the community or a Summerset care home facility. Data collection took place between November 16, 2020, and March 11, 2021. All participants in the study were provided with a NZ $30 (US $21.50) shopping voucher for their involvement in the research.

### Randomization

Participants were randomly allocated to an intervention or waitlist control group by a member of the research team (EB) (1:1 allocation ratio). Simple randomization was performed using a computerized sequence generation software called Research Randomizer. Allocations were concealed in sealed opaque envelopes from the researchers who enrolled participants (KL and IP) until after participants were enrolled and allocated an ID code. At this point, the researcher was deblinded to assign participants to conditions and provide participants with the appropriate instructions. Participants were deblinded after their assignment to conditions.

### Procedure

Once enrolled, participants were contacted over email with instructions for proceeding in the trial. For nursing home residents, their caregiver was copied in the email communications and facilitated the participant’s involvement in the study.

All participants completed an online baseline questionnaire on day 1 of their participation. Then, participants in the intervention group completed a DH training session with a member of the research team for 30 minutes. For all older adults (plus 1 younger adult participant), this took place in-person at their retirement village or nursing home facility. For 7 of 8 younger adults, this took place either in-person at the University of Auckland Clinical Research Centre or online over Zoom video conferencing software (Zoom Video Communications), depending on the lockdown conditions.

All participants received the same technology training, which involved learning how to interact with Bella and completing “day 1” of their intervention week with the researcher present to answer questions. The researcher ensured that the software worked on each participant’s computer. Participants were provided with written instructions and pictures of the user interface that summarized the training session content. For sessions over Zoom videoconferencing, the screen share feature was used and participants received a PDF copy of the interaction instructions. Three participants were trained over Zoom, and 23 participants were trained in-person.

Participants were asked to interact with Bella for at least 15 minutes per day over 1 week. Participants visited Bella’s website independently from their place of residence. The daily 15 minutes could include time spent interacting with Bella and doing therapy activities (eg, a deep breathing exercise). They were asked to prioritize completing the mental health and well-being modules before visiting other content. Participants interacted with Bella at their chosen time of day. Participants were sent a daily text reminder to engage in the intervention and were informed that they could text back to receive technical support.

On day 8, the intervention group finished their intervention week and completed an online postintervention questionnaire. One week later, on day 15, intervention group participants filled out an online follow-up questionnaire.

For participants in the waitlist control group, the order of the procedure was slightly different. Participants in the waitlist group completed an online baseline questionnaire on day 1 and then waited for 1 week. On day 8, waitlist participants completed a second online questionnaire, completed the technology training session, and began their intervention week. On day 15, at the end of their intervention week, waitlist participants completed the postintervention questionnaire.

### Measures

**Figure 3:**
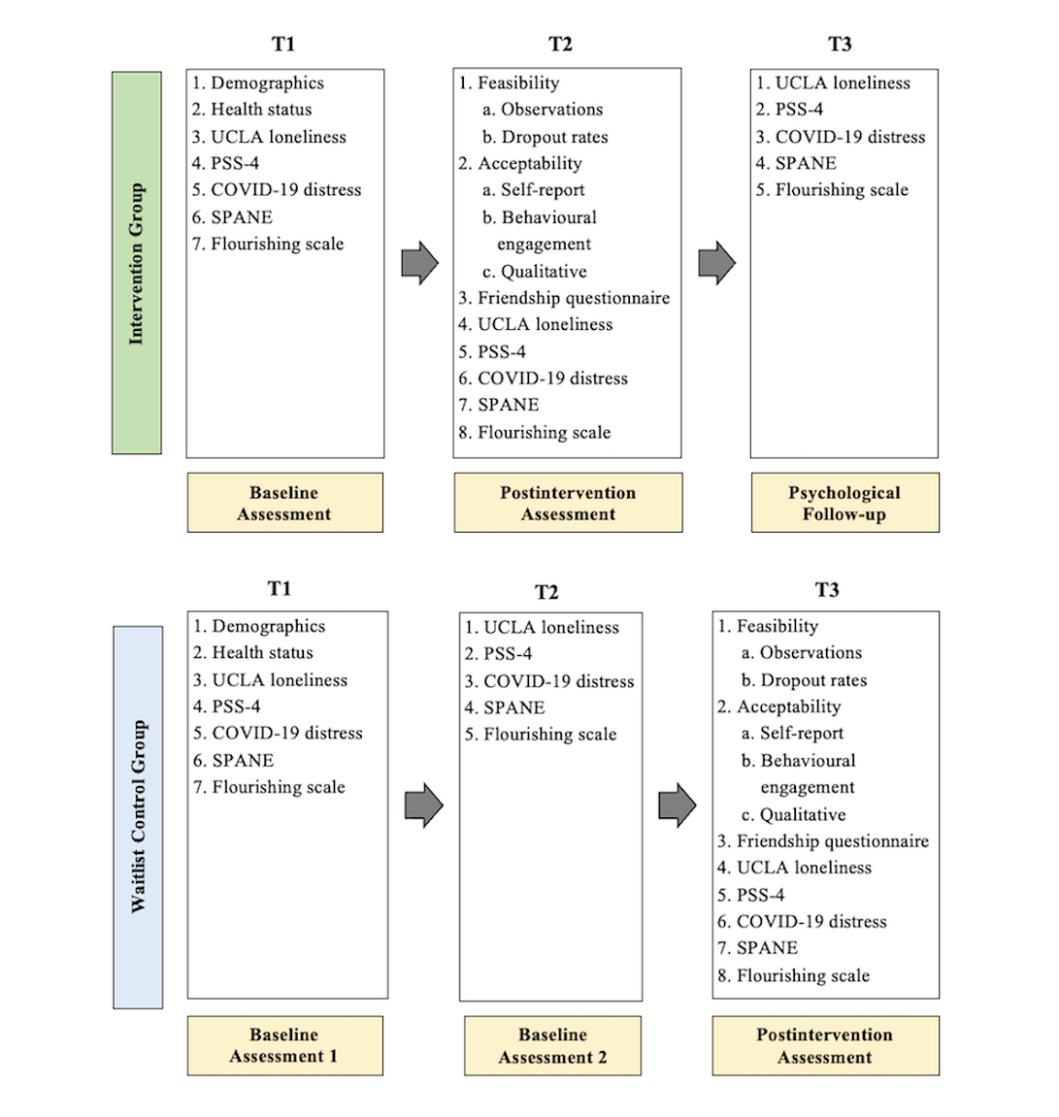
Time points for assessments. PSS-4: Perceived Stress Scale 4 items; SPANE: Scale of Positive and Negative Experiences.

Figure 3 depicts the time points at which each measure was administered. Questionnaires were administered online using Qualtrics, a secure survey website.

#### Feasibility Measures

##### Feasibility of the DH Intervention

Observations were made by a member of the research team (KL) about how the intervention was used (eg, independently or with the aid of a caregiver), along with dropout rates and reasons. Observations were also made regarding the feasibility of the technology training methods for younger and older adults, and nursing home residents. Instances were recorded where participants refused to receive training through a particular delivery method (eg, video calling).

##### Feasibility of the Study Methods

Observations were recorded during recruitment and data collection by a member of the research team (KL). Observations pertained to the success rate of different recruitment strategies for younger and older adult participants, and challenges associated with data collection from the online forms that participants completed independently.

#### Acceptability Measures

##### Acceptability of the DH

Bella’s acceptability was measured using quantitative self-report items and open-ended qualitative questions designed for the study. Behavioral engagement data were also collected. The acceptability measures are outlined in further detail below.

##### Self-Report Items

Participants were asked to rate whether (1) they felt Bella was helpful for promoting resilience and psychological well-being, (2) they felt Bella was helpful for improving feelings of loneliness, and (3) they would be willing to use Bella again in the future, using a 7-point scale with response anchors (1, “definitely no” to 7, “definitely yes”).

##### Qualitative Responses

Participants provided written responses to the following open-ended questions: *What did you like most about Bella?* and *How do you think Bella could be improved?* These questions were intended to provide an overall indication of Bella’s acceptability and to identify aspects of the technology that could be improved.

##### Behavioral Engagement

Behavioral engagement with Bella over 1 week was evaluated by retrospective self-report. Participants reported on which days of the week they visited Bella and estimated approximately how long they used Bella each day in minutes.

##### Acceptability of the Intervention Content

The acceptability of each psychological intervention module was evaluated separately in the postintervention questionnaire. Participants rated how much they liked the brain and stress module, Headspace (if they visited), and the expressing kindness challenge (including each of its 3 activities) on a 7-point scale with response anchors (1, “not at all” to 7, “very much”). Participants rated how beneficial they found the expressing kindness challenge for well-being, and how well they felt the brain and stress module improved their understanding of the stress response on a 7-point scale (1, “not at all” to 7, “very much”). Participants who visited Headspace were asked whether they felt that Headspace was a helpful resource to link to with a dichotomous yes/no response option. The participants were also asked the following qualitative question: *Were there any particular topics that you would have liked to talk about with Bella, which were not available?* Participants provided written responses. Self-reported behavioral engagement data were collected on whether participants visited each module and whether they did the activity that the module asked of them.

#### Rapport With the DH

Rapport with Bella was measured using the 20-item Friendship Questionnaire developed by Johanson et al [53], with items adapted to suit a DH. It is comprised of items taken from multiple friendship scales, including the McGill Friendship Questionnaire, the McGill Friendship Questionnaire Functions scale, the Interactant Satisfaction Survey, and the Acquaintance Description Form-F2 [53]. Participants indicated their agreement with each item using a 5-point Likert scale from 1 (“strongly disagree”) to 5 (“strongly agree”). Responses were summed to derive a total score from 20 to 100, where a higher score indicated greater rapport. The friendship questionnaire has been shown to have good internal consistency reliability when used to evaluate a social robot in a New Zealand adult sample (α=.94) [53]. The scale showed good internal consistency reliability in this study sample when adapted for use with a DH (α=.95). The adapted scale has been included in Multimedia Appendix 1.

#### Loneliness

Loneliness was measured using the 20-item UCLA Loneliness Scale (Version 3) [54]. Participants rated how often they felt the way described in each statement using a 4-point scale. Responses could range from 1 (“never”) to 4 (“always”). Items were reverse coded where appropriate, and responses were summed to derive a total score from 20 to 80. A higher score indicated greater perceived loneliness. This scale was developed with language to improve readability and has demonstrated acceptable psychometric properties with older adults. This includes good internal consistency reliability (α=.89), discriminant validity with social support, and construct validity [54].

#### Psychological Stress

Perceived stress was measured using the 4-item Perceived Stress Scale (PSS-4) [55], which evaluated the degree of stress participants felt over the past week using a 5-point scale (0, “never” to 4, “very often”). Items 2 and 3 were reverse coded, and all responses were summed to form a total score from 0 to 16. A higher score indicated greater perceived stress. Although the psychometric properties of the PSS-10 and PSS-14 have been shown to be superior, the PSS-4 was chosen as it is a shorter measure of perceived stress that reduces participant burden and has adequate internal consistency reliability [56].

#### COVID-19 Distress

Worry about contracting COVID-19 was measured using a 1-item scale [57]. The scale evaluated participants’ degree of worry over the past week on a 4-point scale as follows: 0, “I do not worry about getting COVID-19;” 1, “I occasionally worry about getting COVID-19;” 2, “I spend much of my time worrying about getting COVID-19;” and 3, “I spend most of my time worrying about getting COVID-19.”

#### Positive and Negative Affect

The Scale of Positive and Negative Experiences (SPANE) has two 6-item subscales that measure positive emotions (SPANE-P) and negative emotions (SPANE-N) [58]. The subscales measured the extent to which positive or negative emotions were experienced over the past week using a 5-point scale (1, “very rarely or never” to 5, “very often or always”). For each subscale, responses were summed, and a total score was derived ranging from 6 to 30. A higher score indicated stronger positive or negative affect, depending on the subscale. Affect balance scores (SPANE-B) were calculated, which indicate the participant’s balance of positive and negative affect from −24 to 24, where positive scores indicate more positive than negative affect during the period. The scale has good internal consistency (SPANE-B: α=.89; SPANE-P: α=.87; SPANE-N: α=.81) and convergent validity [58].

#### Psychological Well-Being

Psychological well-being was measured using the 8-item Flourishing Scale [58]. Participants were asked to rate their perceived success across items pertaining to different aspects of psychological well-being, including purpose, relationships, self-esteem, and optimism, using a 7-point Likert scale (1, “strong disagreement” to 7, “strong agreement”). Responses were summed to derive a total well-being score between 8 and 56. Higher scores indicated greater well-being. The Flourishing Scale has been shown to have good psychometric properties including convergent validity and discriminant validity. It has also been shown to have good reliability and validity in a nationally representative New Zealand sample [59].

### Data Analysis

#### Quantitative Data

Data were analyzed using SPSS software (version 27; IBM Corp). Missing data were addressed by imputing the mean score of the participant’s other responses to the scale at the timepoint. For 1-item scales, where it was not possible to impute a score or where the participant did not complete a full scale, the participant’s data were excluded from analysis of the relevant variable.

Baseline demographic and psychological variables were calculated for the overall sample, and compared between groups using chi-square tests and independent samples *t* tests. Average acceptability and rapport scores were calculated for the overall sample, and independent samples *t* tests were conducted to compare group means. A series of mixed factorial analyses of variance (ANOVA) were conducted to evaluate the main and interaction effects of condition and time on psychological outcomes. Data were checked for violations of test assumptions. Greenhouse-Geisser–adjusted values were reported for data where sphericity assumptions were violated (COVID-19 distress, SPANE-P, and SPANE-B). Exploratory pair-wise comparisons with Bonferroni corrections were conducted as follow-up analyses for significant or trending effects.

#### Qualitative Data

Written responses to 3 open-ended questions were analyzed using reflexive thematic analysis [60], which is theoretically flexible and suitable for analyzing the content of language data. One member of the research team (KL) conducted the analysis in keeping with recommendations by Braun & Clarke [60], using the following steps: (1) familiarization with the data, (2) coding, (3) generating initial themes, (4) reviewing themes, (5) defining and naming themes, and (6) writing results. An inductive approach was taken whereby coding and theme development were informed by the content of the data. As part of the theme development in stages 3, 4, and 5, themes and subthemes were checked against the original data set and each other to ensure that they were internally coherent (ie, organized around a clear central concept), consistent, and distinctive. Themes and subthemes were split or combined during the review process (stage 4) to improve specificity. All coded data for each theme and subtheme were collated to assist with result writeup. Data were combined across groups as both received the same intervention.

## Results

### Participants

**Figure 4:**
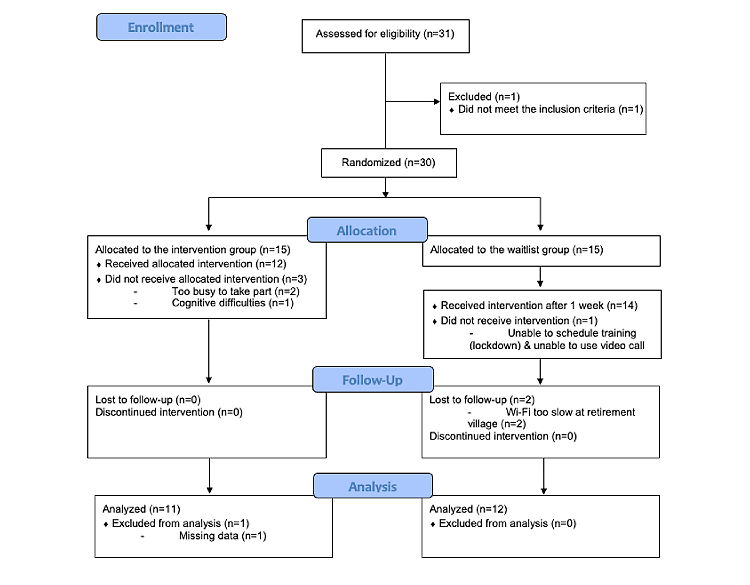
CONSORT (Consolidated Standards of Reporting Trials) diagram of participant flow.

Participants were predominantly female (24/30, 80%) and Caucasian (22/30, 73%), and mainly had high school or less education (14/30, 47%). Half of the sample (15/30, 50%) reported an underlying medical condition. Participants reported low levels of loneliness (mean 37.79, SD 9.90) and stress at baseline (mean 3.86, SD 2.88). Participant characteristics at baseline are reported in Multimedia Appendix 2. A CONSORT diagram depicts participant flow through the study in Figure 4.

### Feasibility of the DH Intervention

#### DH Training Method

Older adults required technology training to be completed in-person at their retirement village with a member of the research team (KL or IP). This method worked well as it avoided any discomfort with using the video calling software. One older adult was offered training over the Zoom video calling software during the lockdown period and refused as she was not able to use the software. Only 1 of 22 older adult participants did not have a webcam as part of their computer, which was uncovered during the technology training (the DH website requires a webcam). To solve this, a webcam was borrowed from the retirement village reception and installed by a member of the research team (IP) during the training session.

Younger adult participants were generally able to be trained either in-person from a clinic room at the university (outside of the lockdown period) or online over Zoom (during the lockdown period). Video calling did not appear to impact the effectiveness of the training. Technical support requests were low for younger adults during the study, irrespective of how their training was delivered. One younger adult participant who was a nursing home resident required in-person technology training.

#### Dropout

Six participants withdrew from the study (all older adults). The reasons for withdrawal were as follows: (1) the Wi-Fi speed at the retirement village location was too slow for Bella to load properly (n=2); (2) cognitive health difficulties interfered with understanding study instructions (n=1); (3) the participant was too busy to take part after enrollment (n=2); and (4) technology training could not be scheduled (n=1).

#### Intervention Completion

Twenty-four participants completed the intervention, 22 of whom completed it independently after training. Two participants (1 younger adult and 1 older adult) who were both nursing home residents required caregiver assistance to access the website and interact with Bella.

### Feasibility of Study Methods

#### Feasibility of Recruitment Methods

The majority of older adult participants were recruited through information sessions held at retirement villages (21/22, 96%). One older adult participant was recruited through an email flyer sent by a village staff member to residents.

For younger adults, the most effective recruitment method was by advertisement to a university junk email list (5/8, 63%). Facebook advertising and word of mouth each resulted in recruitment of 1 participant, and 1 participant was recruited from an information session at a nursing home.

#### Feasibility of Assessment Delivery Methods

Most participants completed assessments online using Qualtrics without significant issue. Two participants reported instances where they were unsure whether their responses had been submitted. Nursing home residents were unable to complete assessments independently on a website and required caregiver assistance.

### Acceptability of the DH

Overall, participants reported that Bella was somewhat helpful for promoting resilience and psychological well-being (mean score 4.39 out of 7, SD 1.83) and for improving any feelings of loneliness (mean score 4.09 out of 7, SD 1.76), as responses on average were above the mid-point. Participants were somewhat willing to use Bella again in the future (mean score 4.09 out of 7, SD 1.98). Younger and older adults rated Bella similarly across the acceptability items, and no significant differences were found.

On average, participants interacted with Bella 6 out of 7 days (mean 6.23, SD 1.19). Participants interacted with Bella for approximately 20 minutes per day (mean 20.20, SD 13.95); 5 minutes longer than the 15 minutes per day requested by the researchers. The average total interaction time with Bella over 1 week was 128 minutes (mean 128.33, SD 102.77). There were no significant differences between younger and older adults in engagement behavior.

Participants identified several strengths and limitations of Bella through responses to the following 2 written open-ended questions: *What did you like most about Bella?* and *How do you think Bella could be improved?* Themes, subthemes, and representative quotes are presented below in Tables 1 and 2. Definitions of themes are presented in Multimedia Appendix 3. Overall, participants liked aspects of Bella’s appearance, speech, and interpersonal skills; the informational support Bella provided; the user experience; and the interaction with a new technology. Aspects of Bella that participants felt could be improved were the human likeness of her interaction behaviors and voice, and aspects of the conversation design (eg, more personalization and conversation topics). Some participants reported that they felt Bella would be improved with gradual advances in the underlying technology (eg, natural language understanding). Other participants reported that they would have preferred to interact with a real human, and some participants did not request any improvements.

**Table 1:** Themes, subthemes, and representative quotes describing what participants liked most about Bella.

Themes and subthemes		Representative quotes
**Bella’s appearance**		
	Facial expressions	*Her friendly smile.* [Participant ID 106]
	Human-like	*I liked the ‘human’ aspects of her.* [Participant ID 124]
	Attractive face	*She is attractive looking.* [Participant ID 112]
	Similarity to user	*I felt in some ways visually represented by Bella.* [Participant ID 124]
**Bella’s speech**		
	Gentle voice	*Quite relaxing. Liked the soft voice. You can hear compassion in her voice.* [Participant ID 115]
	Self-disclosure	*I really appreciated how the conversation would be ‘softened’ by more personal statements from her.* [Participant ID 124]
	Clear language	*Clear speaking.* [Participant ID 118]
**Bella’s interpersonal skills**		
	Companionship	*That she was there.* [Participant ID 117]
	Nonjudgmental	*Feel like you can tell her just about anything and she wouldn’t be shocked. Like talking to a priest in confession.* [Participant ID 115]
	Friendly personality	*Friendly and likeable.* [Participant ID 119]
	Validating	*Nice being told what you’re feeling is normal.* [Participant ID 115]
	Calm personality	*Her calmness.* [Participant ID 125]
**Informational support**		
	Quality resources	*A good selection of resources.* [Participant ID 126]
	Accessible delivery	*Accessibility, most people would find her approachable.* [Participant ID 103]
**User experience**		
	Interaction modalities	*The direct interaction.* [Participant ID 130]
	User controls interaction	*A good medium that allowed me to have plenty of control.* [Participant ID 124]
	Easy to use	*That it was easy to use.* [Participant ID 127]
	Overall experience	*Enjoyed the ‘experience’ of Bella and certainly a helpful person if you were lonely.* [Participant ID 111]
**Novel technology**		
	Something different	*Something to do with somebody to talk to me. She was different.* [Participant ID 104]
	A new technology	*New technology is always intriguing, and I had heard of Bella before.* [Participant ID 105]

**Table 2:** Themes, subthemes, and representative quotes describing what participants felt could be improved about Bella.

Themes and subthemes		Representative quotes
**Interaction behaviors**		
	More human-like movements	*The movement of Bella is still quite robotic and her eyes cannot really focus, which makes her sometimes not seem very engaged in the interaction.* [Participant ID 125]
	Ability to touch	*A sad thing is you can’t touch her. Make a doll out of her. A nice cuddly soft doll.* [Participant ID 115]
	More positive emotional expression	*Smiles.* [Participant ID 118]
**Conversation design**		
	Extra conversation topics	*A wider range of subjects.* [Participant ID 101]
	Greater interactivity	*More interaction by talking to her, rather than just a yes or no.* [Participant ID 107]
	More personalized responses	*Answers need to be more individualized (e.g., welcoming the participant by name and building on each day’s responses).* [Participant ID 112]
	Regularly update information	*Information wasn’t as up to date (eg, COVID levels).* [Participant ID 129]
	Avoid human-like backstories	*I didn’t like the comments she made such as ‘I contacted my friend today.’ I found it weird that she was pretending to be real. I would have preferred if it was just accepted as an interface that had a good selection of resources that you could navigate in an interesting dynamic way.* [Participant ID 126]
	Address user by name	*By addressing each person by their name, that way we could feel in the moment.* [Participant ID 116]
	Incorporate user’s life experience	*Most older people (I am 82) have many years of life experience and perhaps some way could be found to take life experience into consideration.* [Participant ID 112]
**Robotic speech**		
	A more human-like voice	*Perhaps maybe not sound so robotic? It might be hard to achieve but all the inflections and warmth that someone would have in their tone and delivery was missing and I think that’s what would have made Bella more engaging for me.* [Participant ID 127]
	Formal speech delivery	*Improve pronunciation. Use correct English (nope and yeah are not acceptable).* [Participant ID 105]
**Technology advances**		
	Natural language understanding	*I think given advances in technology this will happen anyway. Found the response from her using the audio didn’t always work so found it easier to type the responses to her.* [Participant ID 102]
	General technology advances	*I guess technology will advance and make changes but pretty amazing now.* [Participant ID 111]
Preference for a real human		*Personally, I believe talking to a real person is far more desirable.* [Participant ID 106]
No changes		*I accept it for what she is. Saying that we are not all the same, she is different, she is what she is.* [Participant ID 104]

### Acceptability of Intervention Content

#### The Expressing Kindness Challenge

Of 24 participants, 22 visited the expressing kindness challenge. Overall, the expressing kindness challenge was liked by participants (mean score 5.50 out of 7, SD 1.34), as were the 3 daily challenges of which it was comprised. Participants reported liking reaching out to a friend (mean score 5.95 out of 7, SD 1.13), telling a friend what they appreciate about them (mean score 5.77 out of 7, SD 1.09), and making a gratitude list (mean score 5.71 out of 7, SD 1.14). Participants reported that the expressing kindness challenge felt beneficial for their well-being (mean score 5.00 out of 7, SD 1.95). There were no significant differences between younger and older adults in terms of how much they reported to like the expressing kindness challenge (mean score 5.57, SD 1.39 vs mean score 5.47, SD 1.36; *t*_20_=−0.17; *P*=.87), its activities (all *P*>.27), or how beneficial the module felt for well-being (mean score 5.57, SD 1.13 vs mean score 4.73, SD 2.22; *t*_20_=−0.94; *P*=.36).

The majority of participants (13/24, 59%) visited all 3 tasks of the expressing kindness challenge. Two participants (9%) visited only 2 tasks, and 7 participants (32%) visited only 1 task. Most participants (15/24, 68%) completed the expressing kindness challenge on 3 consecutive days. One participant completed the challenge in 1 day, and 6 participants (27%) completed the challenge in other ways (eg, spread over a week).

Most participants who visited the expressing kindness challenge attempted the activities. All 20 participants who visited day 1 completed the activity (ie, reaching out to a friend). Of 16 participants who visited day 2, 15 completed the activity (ie, telling a friend what they appreciate about them). All 14 participants who visited day 3 did the activity (ie, make a gratitude list).

#### The Brain and Stress Module

Twenty-one participants visited the brain and stress module. On average, participants reported that they liked the brain and stress module (mean score 5.52 out of 7, SD 1.25), and that it improved their understanding of the stress response (mean score 4.90 out of 7, SD 1.61). There were no significant differences in how much younger and older adults liked the brain and stress module (mean score 5.71, SD 1.38 vs mean score 5.43, SD 1.22; *t*_19_=−0.48; *P*=.63) or how helpful they found the module for improving their understanding of stress (mean score 5.43, SD 1.13 vs mean score 4.64, SD 1.78; *t*_19_=−1.06; *P*=.30).

Of 21 participants who visited the brain and stress module, 18 (86%) reported looking at the mental health tips section afterwards to learn about stress management and mental well-being. Additionally, 17 participants (81%) visited Headspace, which is a meditation website that the DH linked to at the end of the brain and stress module [51]. Of these participants, 6 (35%) tried a deep breathing meditation from Headspace. On average, participants reported liking the meditation exercise that they tried (mean score 5.33 out of 7, SD 1.21). There was no significant difference in how much younger and older adults liked the meditation exercise (mean score 5.00, SD 1.41 vs mean score 6.00, SD 0.00; *t*_4_=0.94; *P*=.40). Moreover, 13 participants (77%) agreed that Headspace was a helpful resource for Bella to share.

#### Other Conversation Modules

Participants visited an average of 9.39 (SD 5.23) other modules beyond the expressing kindness challenge and the brain and stress module (ie, the mental health modules that the researchers asked them to complete in particular). There were no significant differences in how many additional modules younger and older adults visited (mean 8.50, SD 6.37 vs mean 9.87, SD 4.69; *t*_21_=0.59; *P*=.56).

#### Module Visit Behavior

Multimedia Appendix 4 depicts how many participants visited each module. The most popular modules were brain and stress, expressing kindness challenge day 1, move your body, do things that bring joy, watch what you consume, and self-care guide. The least popular module was COVID-19: healthline and resources.

#### Requests for Conversation Topics

Seventeen participants responded to the question *Were there any particular topics that you would have liked to talk about with Bella which were not available?* Six participants reported no additional topics, and 11 participants described topics pertaining to physical health, mental health, entertainment, New Zealand, and other areas, as outlined in Multimedia Appendix 5. Representative quotes are not presented as participants tended to list topics.

### Rapport With the DH

Overall, participants reported a reasonable degree of rapport with Bella (mean score 66.92 out of 100, SD 12.63). There was no significant difference in the amount of rapport reported by younger and older adults (mean 68.13, SD 14.86 vs mean 66.31, SD 12.45; *t*_22_=−0.30; *P*=.77).

### Loneliness

There was no significant main effect of time (*F*_2,40_=0.87; *P*=.43; η_p_^2^=0.04) or condition on perceived loneliness (*F*_1,20_=0.87; *P*=.36; η_p_^2^=0.04). There was no significant interaction effect between time and condition on perceived loneliness (*F*_2,40_=0.01; *P*=.99; η_p_^2^=0.00).

### Stress

There was a significant main effect of condition on perceived stress (*F*_1,20_=6.58; *P*=.02; η_p_^2^=0.25). The intervention group reported significantly lower stress overall (mean 2.30, SE 0.77) compared to the waitlist control group (mean 5.09, SE 0.77) (Multimedia Appendix 6). Exploratory pair-wise comparisons revealed that the intervention group reported significantly lower stress compared to the waitlist group at baseline (mean 2.36, SE 0.77 vs mean 5.46, SE 0.77; *F*_1,20_=8.13; *P*=.01; η_p_^2^=0.29) and at T2 (mean 2.36, SE 0.82 vs mean 5.09, SE 0.82; *F*_1,20_=5.47; *P*=.03; η_p_^2^=0.22). There was no significant main effect of time (*F*_2,40_=0.35; *P*=.71; η_p_^2^=0.02) or interaction effect between time and condition on perceived stress (*F*_2,40_=0.13; *P*=.88; η_p_^2^=0.01).

### COVID-19 Distress

There was no significant main effect of time (*F*_1.47,29.44_=0.12; *P*=.83; η_p_^2^=0.01) or condition on COVID-19 distress (*F*_1,20_=0.03; *P*=.41; η_p_^2^=0.00). There was no significant interaction effect between time and condition on COVID-19 distress (*F*_1.47,29.44_=0.83; *P*=.41; η_p_^2^=0.04).

### Positive and Negative Affect

There was no significant main effect of time (*F*_1.44,28.89_=0.93; *P*=.38; η_p_^2^=0.04) or condition on the degree of positive affect reported (*F*_1,20_=0.45; *P*=.51; η_p_^2^=0.02). There was no significant interaction effect between time and condition on positive affect (*F*_1.44,28.89_=0.26; *P*=.70; η_p_^2^=0.01).

There was no significant main effect of time (*F*_2,40_=1.51; *P*=.23; η_p_^2^=0.07) or condition on the degree of negative affect reported (*F*_1,20_=2.50; *P*=.13; η_p_^2^=0.11). There was no significant interaction effect between time and condition on negative affect (*F*_2,40_=1.78; *P*=.18; η_p_^2^=0.08).

There was no significant main effect of time (*F*_1.50,40_=1.03; *P*=.35; η_p_^2^=0.05) or condition on the balance of positive and negative affect reported (*F*_1,20_=1.28; *P*=.27; η_p_^2^=0.06). There was no significant interaction effect between time and condition on the balance of positive and negative affect (*F*_1.50,40_=0.89; *P*=.39; η_p_^2^=0.04).

### Psychological Well-Being

There was a trend toward a significant main effect of condition on psychological well-being (*F*_1,20_=3.44; *P*=.08; η_p_^2^=0.15). The intervention group reported greater well-being overall (mean 49.00, SE 1.80) compared to the waitlist group (mean 44.27, SE 1.80) (Multimedia Appendix 7). Exploratory pair-wise comparisons revealed a trend toward the intervention group reporting greater well-being compared to the waitlist group at baseline (mean 49.27, SE 1.99 vs mean 43.91, SE 1.99; *F*_1,20_=3.64; *P*=.07; η_p_^2^=0.15) and at T3 only (mean 49.46, SE 2.03 vs mean 43.82, SE 2.03; *F*_1,20_=3.84; *P*=.06; η_p_^2^=0.16). There was no significant main effect of time (*F*_2,40_=0.01; *P*=.99; η_p_^2^=0.00) or interaction effect between time and condition on psychological well-being (*F*_2,40_=1.29; *P*=.29; η_p_^2^=0.06).

## Discussion

### Contextualization

Technology has come to play an important role in combatting the COVID-19 pandemic. Artificial intelligence technologies have been rapidly deployed to assist in diagnosing COVID-19 cases and forecasting epidemic development, contact tracing, aiding in drug and vaccine discovery research, and predicting patient outcomes such as disease severity, length of hospital stay, and mortality risk [61,62]. This study proposes that DHs may be an additional technology to aid in health care during the COVID-19 pandemic by providing remote psychological support to people at risk of developing more severe illness. Indeed, other studies have found that digital psychological interventions have been effective during the pandemic (eg, mHealth apps) [63,64].

This study found that a DH was a feasible and acceptable way to deliver a remote loneliness and stress intervention to at-risk older adults living independently and to younger adults with a chronic health condition based on behavioral, qualitative, and some self-report data. The intervention was less feasible for nursing home residents who required caregiver assistance to participate, which may have increased caregiver burden.

Prior to the pandemic, evidence had been building in support of the effectiveness, feasibility, and acceptability of CAs, including embodied agents, at delivering remote psychology interventions and assessments [65,66]. However, their actual adoption in health care settings was low [65], and their efficacy varied depending on the intervention they delivered [66]. Some CAs have technological limitations such as issues with speech recognition, which will be improved as technology advances, but until that stage, these limitations may negatively impact usage intentions [67]. DHs are a new type of CA with an engaging hyperrealistic appearance and neural network-driven behaviors that, prior to this study, had not been evaluated for providing remote loneliness interventions to older adults or adults with chronic health conditions. This study achieved positive results that align with prior CA research showing good acceptability at delivering loneliness interventions to older people [38,68,69], and psychological support for well-being [70] and anxiety in adults with chronic health conditions [39]. 

A challenge of evaluating the effectiveness of CAs in psychology applications is the large heterogeneity of outcome measures, psychological interventions, and technology features across the literature, which makes comparisons difficult, alongside a shortage of RCTs [48,65,66]. This study was conducted in preparation for a larger RCT to investigate intervention effectiveness. In this pilot RCT, the trial methods were found to be feasible, and they support conducting a future RCT. Exploratory analyses of the psychological variables did not reveal any significant effects. However, this is not unexpected as the pilot trial was not powered to detect any significant group differences in psychological outcomes. Furthermore, it is likely that a 1-week intervention is not long enough to see effects on general loneliness.

Questions remain around how to optimally design CAs for health care applications [66,71]. Some research suggests that greater personalization of CAs (eg, through feedback, daily health reports, and recommendations) may improve acceptability and user engagement [72]. Indeed, some participants from this study reported that they would have liked more personalized responses from Bella. Other research has found that a variety of verbal and nonverbal relational behaviors may contribute to better relationships and usage intention with embodied agents [48,71]. However, there may be interaction effects between relational behaviors, user characteristics, and use context [48]. Participants in this study requested more relational behaviors, such as increased positive emotional expression, addressing the user by name, and incorporating the user’s life experience, among others. Incorporating these changes to Bella’s design may help to boost her acceptability scores, alongside gradual developments in her underlying technology. Indeed, other research has argued the importance of a co-design process with users and stakeholders to increase acceptability and encourage successful implementation, and future research should adopt this process [73,74].

### Strengths and Limitations

This study investigated a novel application of DH technology and adopted a pilot RCT design to inform the methodology of future trials. However, there were several methodological limitations. A sample bias may have occurred whereby participants who volunteered may have been more digitally literate or comfortable with using novel technologies. Moreover, the sample predominantly included Caucasian women; therefore, it is unclear how well the results would generalize to a more diverse population. Even though randomization was conducted, there were significant group differences at baseline in stress, and a larger sample likely would have eliminated these differences. Changes in and out of lockdown conditions in Auckland during the data collection period could have affected the psychological results and degree of engagement in the study. Moreover, there was no control for the psychological follow-up data of our waitlist group, and this should be addressed in a future trial. It is also unclear what the level of engagement with the DH would be outside of a clinical trial context. Research has shown that engagement with eHealth interventions is often lower than what is observed in trials [23,24].

### Future Research

The results suggest several directions for future research. A fully powered RCT should investigate the effects of the DH intervention on loneliness and stress. This trial could address the methodological limitations of our study. An active control condition (eg, a chatbot and a website) could be used to provide stronger evidence of effectiveness and reduce the chance that outcome improvements are due to confounding variables (eg, passage of time and researcher attention). The length of follow-up for psychological measures should be extended, along with the length of the intervention. Many loneliness interventions take place over 4 to 6 months with weekly sessions that take an hour or more [16]. The intervention content could be expanded with evidence-based techniques, such as cognitive behavioral and mindfulness exercises to reduce maladaptive social cognition, which have been shown to be the most effective techniques for reducing loneliness in a meta-analysis [16,19,75]. Additionally, the conversation topics that participants requested (eg, physical health support and entertainment) and their feedback should be incorporated to increase acceptability. Other methodological changes for a future RCT could include using other recruitment strategies to achieve a more diverse sample that is more representative of the general population. A future trial could also change the eligibility criteria to require a moderate or high loneliness score. Individuals with higher loneliness at baseline may find the activities more beneficial for well-being and may have more room to improve their loneliness scores. Intervention effectiveness could be investigated separately in younger and older adults. This would allow for tailoring of the intervention content to the age group (with age-appropriate activities, examples, and conversation topics), as well as adapting the DH’s design to be more similar to the user population (eg, older adults could interact with an older DH). Moreover, separate trials would allow for more streamlined processes for recruitment and technology training. Lastly, future research could examine DHs in other therapeutic applications and in more diverse patient populations. More research is also needed to discern how DHs in psychology applications should be designed to maximize acceptability and engagement.

### Conclusion

Bella, a DH, was found to be a feasible and acceptable way to deliver a remote loneliness intervention to at-risk adults facing social restrictions during the COVID-19 pandemic, based on behavioral, qualitative, and some self-report data. The results support conducting a larger and longer RCT to investigate intervention effectiveness, and indicate that several changes should be made to the technology, intervention content, and trial design. DHs are a novel technological solution that may provide remote psychological support to socially restricted at-risk groups during pandemics. Research should examine the use of DHs in other health care applications with diverse patient populations.

## Appendix

Multimedia Appendix 1 The Friendship Questionnaire (Johanson et al, 2020 [<xref ref-type="bibr" rid="ref53">53</xref>]) adapted to the digital human.

Multimedia Appendix 2 Participant characteristics at baseline.

Multimedia Appendix 3 Definitions of themes in response to the qualitative questions.

Multimedia Appendix 4 Number of participants who visited each module.

Multimedia Appendix 5 Conversation topics that participants would like to talk about with Bella.

Multimedia Appendix 6 Perceived stress (mean scores) between groups over the 3 time points.

Multimedia Appendix 7 Psychological well-being (mean scores) between groups across the 3 time points.

Multimedia Appendix 8 CONSORT-eHEALTH checklist (V 1.6.1).

## Abbreviations

CA: conversational agent
DH: digital human
MMSE: Mini-Mental State Examination
PSS: Perceived Stress Scale
RCT: randomized controlled trial
SPANE: Scale of Positive and Negative Experiences
