# Supplementary material for: A Digital Human for Delivering a Remote Loneliness and Stress Intervention to At-Risk Younger and Older Adults During the COVID-19 Pandemic: Randomized Pilot Trial
Source: JMIR Ment Health. 2021 Nov 8;8(11):e31586. doi: 10.2196/31586 (PMC8577546; doi:10.2196/31586)
Supplement: Multimedia Appendix 1 [file mental_v8i11e31586_app1.docx]

**Multimedia Appendix 1**

*The Friendship Questionnaire (Johanson et al., 2020) adapted to the digital human.*

|  | **Strongly disagree** | **Disagree** | **Neutral** | **Agree** | **Strongly agree** |
| --- | --- | --- | --- | --- | --- |
| I enjoyed talking with Bella |  |  |  |  |  |
| Bella helped me with any feelings of loneliness |  |  |  |  |  |
| Bella shared her emotions with me |  |  |  |  |  |
| I would trust Bella to use if I needed some support again |  |  |  |  |  |
| Bella was supportive of my needs |  |  |  |  |  |
| I felt comfortable interacting with Bella |  |  |  |  |  |
| I felt I would be able to tell private and sensitive things to Bella |  |  |  |  |  |
| Bella is genuinely likeable |  |  |  |  |  |
| I felt Bella expressed feelings and emotions that were appropriate for the situation |  |  |  |  |  |
| I feel like Bella could potentially be a friend of mine, in a way |  |  |  |  |  |
| I would be happy to interact with Bella again |  |  |  |  |  |
| Bella’s answers were helpful to me |  |  |  |  |  |
| Bella showed some sensitivity towards my emotions |  |  |  |  |  |
| I think Bella would be loyal |  |  |  |  |  |
| I felt confident interacting with Bella |  |  |  |  |  |
| Bella’s support was useful to me |  |  |  |  |  |
| I think Bella would make me feel more calm if I was nervous |  |  |  |  |  |
| Bella seemed to enjoy helping me |  |  |  |  |  |
| I felt like Bella was being true and sincere |  |  |  |  |  |
| I would like to have a friendly chat with Bella |  |  |  |  |  |
