# Supplementary material for: A Digital Human for Delivering a Remote Loneliness and Stress Intervention to At-Risk Younger and Older Adults During the COVID-19 Pandemic: Randomized Pilot Trial
Source: JMIR Ment Health. 2021 Nov 8;8(11):e31586. doi: 10.2196/31586 (PMC8577546; doi:10.2196/31586)
Supplement: Multimedia Appendix 2 [file mental_v8i11e31586_app2.docx]

**Multimedia Appendix 2**

*Participant characteristics* *at baseline.*

|  | Total | Condition | | Participant type | |
| --- | --- | --- | --- | --- | --- |
|  |  | Intervention | Waitlist | Younger adult | Older adult |
| Participants (*N*) | 30 | 15 | 15 | 8 | 22 |
| Age (*M, SD*) | 68.20 (18.95) | 72.67 (13.79) | 63.73 (22.60) | 40.50 (15.11) | 78.27 (4.61) |
| Gender (Female *n*, %) | 24 (80%) | 10 (67%) | 14 (93%) | 7 (88%) | 17 (77%) |
| Ethnicity: |  |  |  |  |  |
| European (*n*, %) | 22 (73%) | 10 (66%) | 12 (79%) | 4 (50%) | 18 (82%) |
| Māori (*n*, %) | 1 (3%) | 0 (0%) | 1 (7%) | 1 (12.5%) | 0 (0%) |
| Pacific Peoples (*n*, %) | 2 (7%) | 1 (7%) | 1 (7%) | 1 (12.5%) | 1 (18%) |
| Asian (*n*, %) | 2 (7%) | 1 (7%) | 1 (7%) | 2 (25%) | 0 (0%) |
| Education level: |  |  |  |  |  |
| High school or less (*n*, %) | 14 (47%) | 4 (27%) | 10 (66%) | 2 (25%) | 12 (55%) |
| Trade qualification (*n*, %) | 5 (17%) | 4 (27%) | 1 (7%) | 2 (25%) | 3 (14%) |
| Undergraduate degree (*n*, %) | 5 (17%) | 3 (20%) | 2 (13%) | 1 (12.5%) | 4 (18%) |
| Postgraduate degree (*n*, %) | 3 (10%) | 1 (7%) | 2 (13%) | 3 (37.5%) | 0 (0%) |
| Marital status: |  |  |  |  |  |
| Single (*n*, %) | 2 (7%) | 1 (7%) | 1 (7%) | 1 (12%) | 1 (5%) |
| Relationship (*n*, %) | 1 (3%) | 0 (0%) | 1 (7%) | 1 (12%) | 0 (0%) |
| Married/ living with partner (*n*, %) | 11 (37%) | 7 (46%) | 4 (27%) | 4 (50%) | 7 (32%) |
| Separated/ divorced (*n*, %) | 2 (7%) | 1 (7%) | 1 (7%) | 1 (12%) | 1 (5%) |
| Widowed (*n*, %) | 11 (37%) | 3 (20%) | 8 (52%) | 1 (12%) | 10 (45%) |
| Work status: |  |  |  |  |  |
| Full-time (*n*, %) | 2 (7%) | 1 (7%) | 1 (7%) | 2 (25%) | 0 (0%) |
| Part-time (*n*, %) | 2 (7%) | 1 (7%) | 1 (7%) | 2 (25%) | 0 (0%) |
| Retired (*n*, %) | 20 (67%) | 10 (66%) | 10 (66%) | 1 (12%) | 19 (86%) |
| Unemployed (*n*, %) | 3 (10%) | 0 (0%) | 3 (20%) | 3 (38%) | 0 (0%) |
| Residence type: |  |  |  |  |  |
| Care home (*n*, %) | 4 (13.3%) | 4 (27%) | 0 (0%) | 1 (12%) | 3 (14%) |
| Independent living at retirement  village (*n*, %) | 19 (63.3%) | 9 (60%) | 10 (66%) | 0 (0%) | 19 (86%) |
| Community (*n*, %) | 7 (23.3%) | 2 (13%) | 5 (33%) | 7 (88%) | 0 (0%) |
| Health: |  |  |  |  |  |
| Underlying medical condition (*n*, %) | 15 (50%) | 8 (53%) | 7 (47%) | 8 (100%) | 7 (32%) |
| Medical condition type: |  |  |  |  |  |
| Serious heart condition (*n*, %) | 4 (13.3%) | 3 (20%) | 1 (7%) | 0 (0%) | 4 (18%) |
| Immunocompromised condition (*n*, %) | 7 (23.3%) | 2 (13%) | 5 (33%) | 6 (75%) | 1 (5%) |
| Diabetes (*n*, %) | 2 (6.7%) | 2 (13%) | 0 (0%) | 0 (0%) | 2 (9%) |
| BMI of 40 or higher (*n*, %) | 1 (3.3%) | 0 (0%) | 1 (7%) | 1 (12.5%) | 0 (0%) |
| Pregnancy third trimester stage (*n*, %) | 1 (3.3%) | 1 (7%) | 0 (0%) | 1 (12.5%) | 0 (0%) |
| Not applicable (*n*, %) | 15 (50%) | 6 (47%) | 8 (53%) | 0 (0%) | 15 (68%) |
| Immunocompromised condition: |  |  |  |  |  |
| Immune deficiency (*n*, %) | 3 (43%) | 1 (50%) | 1 (20%) | 3 (38%) | 0 (0%) |
| Immune weakening medication use (*n*, %) | 2 (29%) | 0 (0%) | 2 (40%) | 2 (25%) | 0 (0%) |
| Cancer treatment (*n*, %) | 1 (14%) | 1 (50%) | 2 (40%) | 1 (13%) | 0 (0%) |
| MMSE (*M, SD*) | 28.18 (1.59) | 27.83 (1.80) | 28.60 (1.27) | - | - |
| Psychological: |  |  |  |  |  |
| UCLA Loneliness (*M, SD*) | 37.79 (9.90) | 36.62 (11.01) | 38.80 (9.09) | 44.00 (10.47) | 35.30 (8.74) |
| PSS4 (*M, SD*) | 3.86 (2.88) | 2.69 (2.29) | 4.87 (3.02) | 5.75 (2.44) | 3.10 (2.73) |
