# Supplementary material for: A Digital Human for Delivering a Remote Loneliness and Stress Intervention to At-Risk Younger and Older Adults During the COVID-19 Pandemic: Randomized Pilot Trial
Source: JMIR Ment Health. 2021 Nov 8;8(11):e31586. doi: 10.2196/31586 (PMC8577546; doi:10.2196/31586)
Supplement: Multimedia Appendix 3 [file mental_v8i11e31586_app3.docx]

**Multimedia Appendix 3**

*Definitions of themes in response to the qualitative questions*

| *“What did you like most about Bella?”* | |
| --- | --- |
| Theme | Definition |
| Bella’s appearance | Bella’s facial features and/or humanlikeness. |
| Bella’s speech | Bella’s vocal qualities and speech content. |
| Bella’s interpersonal skills | Bella’s social qualities and feelings of companionship. |
| Informational support | The quality of informational resources that Bella provided and the way in which the resources were delivered. |
| User experience | Specific aspects of the user experience, including the interaction modalities, design, and overall experience. |
| Novel technology | Interacting with a new technology. |
| *“How do you think Bella could be improved?”* | |
| Theme | Definition |
| Interaction behaviours | More humanlike movements and facial behaviours during conversations, as well as the ability to touch the digital human. |
| Conversation design | A richer conversational experience with greater personalisation, regularly updated information, and less humanlike backstories from the digital human. |
| Robotic speech | More humanlike speech prosody and pronunciation. |
| Technology advances | Gradual improvements in the underlying technology, including natural language understanding. |
| Preference for a real human | The participant would have preferred to interact with a real person. |
| No changes | The participant did not request any improvements. |
