# Supplementary material for: A Digital Human for Delivering a Remote Loneliness and Stress Intervention to At-Risk Younger and Older Adults During the COVID-19 Pandemic: Randomized Pilot Trial
Source: JMIR Ment Health. 2021 Nov 8;8(11):e31586. doi: 10.2196/31586 (PMC8577546; doi:10.2196/31586)
Supplement: Multimedia Appendix 4 [file mental_v8i11e31586_app4.docx]

**Multimedia Appendix 4**

*Number of participants who visited each module*

| Module name | *N* |
| --- | --- |
| EK day one | 20 |
| EK day two | 16 |
| EK day three | 14 |
| Brain and stress | 21 |
| Deep breathing | 17 |
| Find connection | 17 |
| Acknowledge your feelings | 17 |
| Watch what you consume | 18 |
| Move your body | 19 |
| Do things that bring joy | 19 |
| Self-care guide | 18 |
| COVID-19: NZ alert levels | 14 |
| COVID-19: Arriving back in NZ | 8 |
| COVID-19: About the virus | 7 |
| COVID-19: Symptoms & prevention | 7 |
| COVID-19: What to do if I’m exposed | 7 |
| COVID-19: Treatment & immunization | 7 |
| COVID-19: Healthline & resources | 6 |
| B&E: Remote work | 13 |
| B&E: Founder mental health | 13 |
| B&E: Interviews with Kiwi leaders | 13 |
| B&E: Business support organisations | 13 |

*Note.* B&E: Business & Entrepreneurship
