# Supplementary material for: A Digital Human for Delivering a Remote Loneliness and Stress Intervention to At-Risk Younger and Older Adults During the COVID-19 Pandemic: Randomized Pilot Trial
Source: JMIR Ment Health. 2021 Nov 8;8(11):e31586. doi: 10.2196/31586 (PMC8577546; doi:10.2196/31586)
Supplement: Multimedia Appendix 5 [file mental_v8i11e31586_app5.docx]

**Multimedia Appendix 5**

*Conversation topics that participants would like to talk about with Bella*

| Themes | Subthemes |
| --- | --- |
| Physical health information | Information about sleep, information about common medical issues for older adults, tips on communicating with doctors, information on when to take medicines, assistance with hospital navigation, mobility pass information |
|  |  |
| Mental health information and exercises | Information on coping with anxiety and uncertainty, information on maintaining work-life balance, completing the mental health tips as exercises with Bella |
|  |  |
| Entertainment | Music, laughter |
|  |  |
| New Zealand | Current events, local information |
|  |  |
| Other | “Lots” (non-specific) |
