# Supplementary material for: A Digital Human for Delivering a Remote Loneliness and Stress Intervention to At-Risk Younger and Older Adults During the COVID-19 Pandemic: Randomized Pilot Trial
Source: JMIR Ment Health. 2021 Nov 8;8(11):e31586. doi: 10.2196/31586 (PMC8577546; doi:10.2196/31586)
Supplement: Multimedia Appendix 6 [file mental_v8i11e31586_app6.docx]

**Multimedia Appendix 6**

*Perceived stress (mean scores) between groups over the three time points*


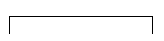

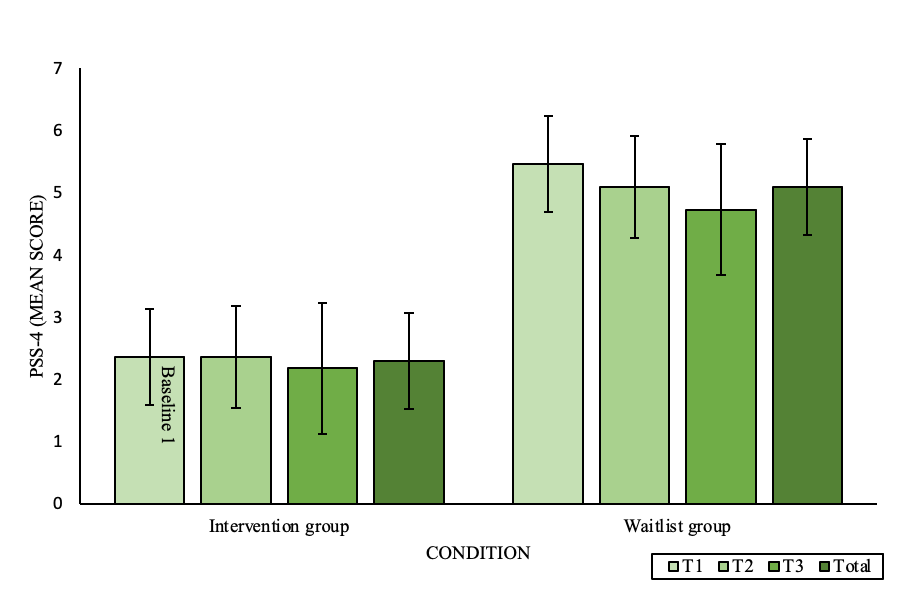


Total

Total

Follow-up

Post-intervention

Post-intervention

Baseline 1

Baseline 2

*

*Note.* Standard error bars are depicted (**p* <.05). Arrows depict when each group took part in the intervention.
