# Supplementary material for: A Digital Human for Delivering a Remote Loneliness and Stress Intervention to At-Risk Younger and Older Adults During the COVID-19 Pandemic: Randomized Pilot Trial
Source: JMIR Ment Health. 2021 Nov 8;8(11):e31586. doi: 10.2196/31586 (PMC8577546; doi:10.2196/31586)
Supplement: Multimedia Appendix 7 [file mental_v8i11e31586_app7.docx]

**Multimedia Appendix 7**

*Psychological well-being (mean scores) between groups across the three time points.*


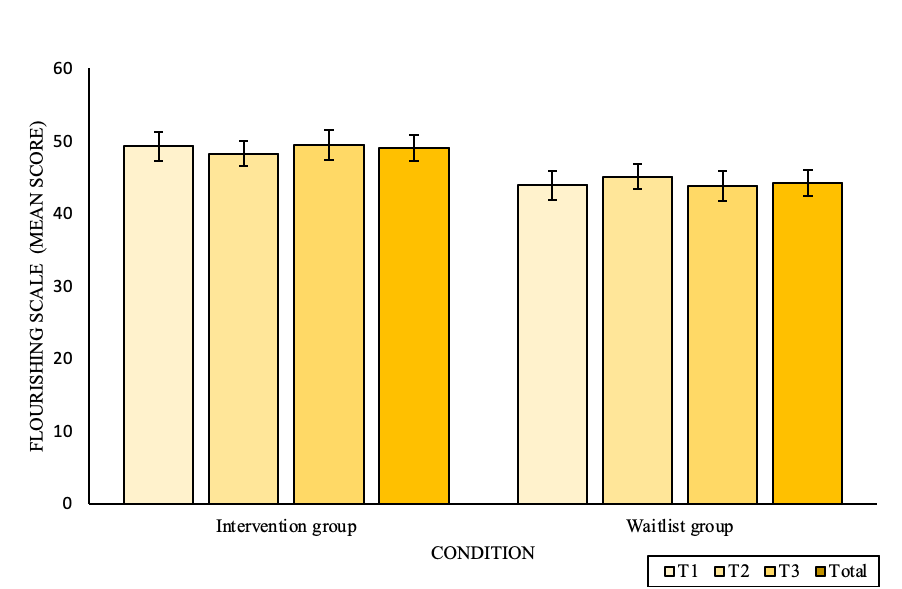


Total

Total

Baseline 2

Follow-up

Post-intervention

Post-intervention

Baseline 1

Baseline 1

*Note.* Standard error bars are displayed. Arrows depict when each group took part in the intervention.
